# Preconception Folate Status, Dietary Intake, Supplementation, and Time to Pregnancy Among Women in a Premarital Health Examination Cohort

**DOI:** 10.3390/nu18142238

**Published:** 2026-07-09

**Authors:** Lirong Nie, Tian Tian, Xiaoqian Jia, Xinyue Lin, Lingzhang Wang, Yuwei Wu, Yali Zhang, Le Zhang, Jianting Li, Zhiwen Li, Zhizhen Liu, Jufen Liu

**Affiliations:** 1 Institute of Reproductive and Child Health, National Health Commission Key Laboratory of Reproductive Health, School of Public Health, Peking University, Beijing 100191, China; nielirong@bjmu.edu.cn (L.N.); jiaxq@pku.edu.cn (X.J.); linxinyue@bjmu.edu.cn (X.L.); 2110306108@stu.pku.edu.cn (L.W.); wuyuwei@stu.pku.edu.cn (Y.W.); zhangyl@bjmu.edu.cn (Y.Z.); zhangle@bjmu.edu.cn (L.Z.); lizw@bjmu.edu.cn (Z.L.); 2 Department of Epidemiology and Biostatistics, School of Public Health, Peking University, Beijing 100191, China; 3 Center for Reproductive Medicine, Department of Obstetrics and Gynecology, Peking University Third Hospital, Beijing 100191, China; tiantianpku@126.com; 4 State Key Laboratory of Female Fertility Promotion, Peking University, Beijing 100191, China; 5 Shanxi Key Laboratory of Birth Defect and Cell Regeneration, Department of Biochemistry and Molecular Biology, Shanxi Medical University, Taiyuan 030001, China; lijianting2021@sxmu.edu.cn; 6 Key Laboratory of Coal Environmental Pathogenicity and Prevention, Ministry of Education, Shanxi Medical University, Taiyuan 030001, China

**Keywords:** preconception folate status, dietary folate intake, folic acid supplementation, time to pregnancy, prospective cohort

## Abstract

**Background/Objectives**: Folate plays a key role in one-carbon metabolism and may influence reproductive function, but evidence regarding its association with fecundability remains inconsistent. This study examined the association of folic acid (FA) supplementation, dietary folate intake, and folate status with time to pregnancy (TTP) among women planning to conceive. **Methods**: This study is a secondary analysis of a prospective cohort study included women enrolled during premarital health examinations in two counties in Shanxi Province (2009–2012). Folate-related exposures were assessed at baseline and included FA supplementation, dietary folate intake, and red blood cell (RBC) folate concentrations. Discrete-time proportional hazards model was used to estimate fecundability ratios (FRs) and 95% confidence intervals (CIs). **Results**: A total of 1475 women without a history of previous pregnancy were included, among whom 24 (1.6%) reported FA supplementation at baseline. Higher intake of several food groups was associated with increased RBC folate concentrations. In separate models, only seafood intake (1–3 times per week) was significantly associated with higher fecundability (FR: 1.23, 95% CI: 1.04, 1.44). In joint models, FA supplementation was associated with higher fecundability at 6 months (FR: 2.07, 95% CI: 1.01, 3.77), 12 months (FR: 1.91, 95% CI: 1.00, 3.30), and 24 months (FR: 1.91, 95% CI: 1.00, 3.30). **Conclusions**: Preconception FA supplementation showed a potential positive association with fecundability when considered alongside dietary intake and folate status, although the evidence was limited by the low use of supplementation. Further studies are needed to clarify the role of folate in human fecundability.

## 1. Introduction

Current studies have suggested that infertility remains a significant global public health concern. Approximately 8–12% of couples worldwide are affected, with prevalence reaching up to 30% in certain regions [1]. Time to pregnancy (TTP) is widely used as an indicator of fecundability, with a longer TTP reflecting a lower probability of conception and impaired reproductive potential [2]. Recent evidence from the United States suggests that TTP has increased over time, particularly among parous women and women older than 30 years [3]. Prolonged TTP can also impose substantial psychological stress on couples and negatively affect marital relationships.

Age is widely recognized as the most important determinant of TTP. Previous studies have reported an inverted U-shaped association between age and fertility, with peak reproductive potential occurring between 20 and 35 years [4,5]. Beyond age, multiple factors have been associated with TTP, including occupational exposures, history of pregnancy, body mass index (BMI), smoking, sleep patterns, work-related stressors, and metabolic syndrome [6,7,8,9,10,11,12,13]. Micronutrient status has also attracted increasing attention as a potentially modifiable determinant of reproductive function. A meta-analysis has reported that antioxidant supplementation may improve fertility in subfertile women and higher intakes of antioxidants such as β-carotene, vitamin C, and vitamin E have been linked to shorter TTP in couples undergoing infertility treatment [14,15].

Folate (vitamin B9) is an essential micronutrient during the preconception period and early pregnancy [16]. Folic acid (FA) supplementation is well-established in reducing the risk of neural tube defects (NTDs) and other adverse pregnancy outcomes [17]. Current guidelines recommend that women planning pregnancy consume 400 μg of FA daily from the preconception period through early pregnancy to ensure adequate folate status [18]. In addition, folate is a key component of one-carbon metabolism and is required for DNA synthesis, repair, and methylation [19]. Through these biological functions, adequate folate status may contribute to reproductive health and support successful conception [20]. However, evidence regarding folate and fecundability remains inconsistent. Prospective studies from Singapore and Denmark reported higher fecundability among women using FA supplements [21,22]. In contrast, other studies have observed no significant relationship between folate status and fecundability. These discrepancies may be partly explained by differences in study populations. For instance, some studies were conducted among folate-replete women with previous pregnancy loss [23], while in most other populations, FA supplementation is common, where the effect of folate may be less pronounced [24].

Given the inconsistent findings, a comprehensive evaluation of folate exposure is needed. Most previous studies have focused on a single dimension, such as supplementation or biomarker levels, which may not fully capture overall folate exposure. Therefore, this study aimed to explore the associations of FA supplementation, dietary folate intake, and folate status with TTP using data from a premarital examination cohort in northern China, with the goal of providing evidence to inform preconception health guidance and improve female fecundability.

## 2. Materials and Methods

### 2.1. Study Design

This study was based on a prospective cohort that was originally established to estimate infertility rates. Participants were recruited from women attending premarital health examinations at maternal and child health care centers in Xiyang County and Shouyang County, Shanxi Province, northern China, between November 2009 and July 2012. Eligible participants were women who were not pregnant at enrollment, intended to conceive within the following 12 months, planned to deliver locally, and provided informed consent. Women with a history of previous pregnancy, those who were already pregnant before marriage, and those without pregnancy intention were excluded from this study. The original cohort enrolled 2302 women, and after applying the eligibility criteria, 1475 women were included in the present analysis.

### 2.2. Assessment of Folate

In this premarital examination cohort, folate-related exposures were comprehensively assessed from three dimensions, including FA supplementation, dietary intake frequency, and biomarker-based folate status. All exposure information was collected at baseline before conception and prior to the initiation of follow-up.

Information on FA supplementation was obtained using a structured questionnaire, which assessed whether participants had used any FA-containing supplements during the past month. This variable was analyzed as a binary indicator (non-use vs. use).

Dietary folate intake was evaluated using a food frequency questionnaire covering the previous three months. From the surveyed food items, those included in the dietary folate frequency score were selected based on both nutritional relevance and statistical association with red blood cell (RBC) folate levels. For each food, intake frequency was classified into four categories: <1, 1–3, 4–6, or ≥7 times per week, corresponding to scores of 1 to 4, respectively. The overall dietary folate frequency score was calculated by summing the scores across all included food items, with higher scores indicating more frequent consumption of folate-rich foods.

Fasting venous blood samples were collected at recruitment. Plasma folate concentrations were measured using a microbiological assay. RBC folate concentrations were subsequently estimated using a previously validated equation based on the linear relationship between the natural logarithms of plasma and RBC folate (ln[RBC folate] = 0.54 × ln[plasma folate] + 4.85; R^2^ = 0.51, *p* < 0.0001) [25,26]. According to established cut-off values based on homocysteine-related metabolic criteria, RBC folate was categorized as deficiency (<340 nmol/L) or non-deficiency (≥340 nmol/L) [27].

### 2.3. Assessment of Covariates

Covariates included age at marriage (<25, 25–29, 30–34, and ≥35 years), BMI (<18.5, 18.5–23.9, and ≥24 kg/m^2^), based on the criteria recommended for Chinese adults, education (junior high school or below/high school or above), occupation (farmer, worker, and other), passive smoking exposure (no/yes), and residence (rural/urban).

### 2.4. Outcome Assessment

Participants were followed until conception or the end of available follow-up, resulting in varying follow-up durations across individuals. Among women who conceived, TTP was defined as the interval between initiation of attempts to conceive and the date of conception. For those who did not conceive, TTP was defined as the duration from initiation of pregnancy attempts to the last follow-up. Participants who did not conceive were treated as censored observations. For analysis, TTP was measured in discrete monthly intervals. Each participant contributed person-month observations until conception or censoring.

### 2.5. Statistical Analysis

Baseline characteristics were summarized as frequencies and percentages for categorical variables. Pregnancy rates within one year and two years were calculated according to categories of RBC folate status.

To identify key dietary contributors to folate status, multivariable linear regression models were fitted with log-transformed RBC folate concentration as the dependent variable. Results were expressed as percentage differences. *p* values were adjusted using the Benjamini–Hochberg false discovery rate (FDR) method.

Discrete-time proportional hazards models were implemented using a complementary log-log link logistic regression to estimate fecundability ratios (FRs) and 95% confidence intervals (CIs). FR represents the probability of conception in a given cycle among exposed versus unexposed individuals. An FR greater than 1 indicates higher fecundability (shorter time to pregnancy), whereas an FR less than 1 indicates reduced fecundability.

To explore potential time-dependent associations, additional analyses were conducted by administratively censoring follow-up at 6, 12, and 24 months. Participants who conceived beyond each time window were treated as censored at the corresponding cutoff.

Folate-related exposures were evaluated using a stepwise modeling strategy. First, each exposure, including FA supplementation, dietary folate intake, and RBC folate levels, was entered separately into multivariable models to estimate its independent association with fecundability. Subsequently, joint models were constructed to simultaneously include multiple exposure components. Model 1 included FA supplementation and the dietary folate frequency score. Model 2 further incorporated RBC folate levels. All models were adjusted for covariates, including age group, BMI group, residence, education, occupation, and passive smoking exposure. Follow-up time was additionally controlled for by including categorical time intervals in the models.

All statistical analyses were performed using R software (version 4.5.1). A two-sided *p* < 0.05 was considered statistically significant.

## 3. Results

A total of 1475 women were included in the analysis (Table 1). The majority were under 25 years old (69.2%), and 65.2% had a normal BMI (18.5–24 kg/m^2^). Over half had an education level of junior high school or below (58.8%), and 49.2% were farmers. Passive smoking exposure was reported by 38.6% of participants, and 69.3% resided in rural areas. Active smoking among women was uncommon in this cohort (0.7%). Notably, only 24 women (1.6%) reported FA supplementation at baseline, indicating that preconception supplementation was uncommon in this population during the study period.

Pregnancy rates according to RBC folate status are presented in Table 2. Within one year, 87.0% of women with RBC folate deficiency became pregnant, compared with 92.4% among those without deficiency (*p* = 0.045). At two years, the corresponding rates were 88.7% and 95.0% (*p* = 0.006).

Table 3 shows the associations between intake frequency of folate-rich foods and RBC folate concentrations. Fruit intake showed consistent positive associations, with increases of 12.77% (95% CI: 7.03, 18.83) for 4–6 times per week and 8.01% (95% CI: 3.38, 12.85) for ≥7 times per week, both remaining significant after FDR correction. Higher intake of root vegetables (≥7 times/week) and nuts (1–3 times/week) was also associated with increased RBC folate levels, with estimated increases of 9.42% (95% CI: 2.91, 16.35) and 6.73% (95% CI: 2.30, 11.35), respectively. Moderate intake of corn and seafood showed positive but non-significant associations after multiple comparison adjustment. No consistent associations were observed for other food groups.

In separate multivariable discrete-time models (Table 4), few individual exposures showed clear associations with fecundability. FA supplementation was not significantly associated with higher fecundability (overall FR = 1.27, 95% CI: 0.79, 1.93). Seafood intake of 1–3 times per week was associated with an FR of 1.23 (95% CI: 1.04, 1.44) overall, compared with intake less than once per week. No significant associations were observed for other foods, or the dietary folate frequency score. RBC folate non-deficiency (≥340 nmol/L) was also not significantly associated with fecundability (overall FR = 1.14, 95% CI: 0.93, 1.41).

Joint models simultaneously including multiple folate-related exposures are shown in Table 5. In Model 1, which included FA supplementation and the dietary folate frequency score, FA supplementation was associated with a higher FR within the first 6 months (FR = 1.73, 95% CI: 1.00, 2.76), although the overall estimate did not reach statistical significance (FR = 1.49, 95% CI: 0.92, 2.27). The dietary folate score was not associated with fecundability. In Model 2, which additionally included RBC folate status, the point estimates for FA supplementation were further increased. The FRs were 2.07 (95% CI: 1.01, 3.77) within 6 months, 1.91 (95% CI: 1.00, 3.30) within 12 months and within 24 months, with an overall FR of 1.89 (95% CI: 0.99, 3.27). RBC folate non-deficiency was not significantly associated with fecundability in this joint model (overall FR = 1.13, 95% CI: 0.92, 1.41).

## 4. Discussion

In this prospective cohort study of 1475 women without prior pregnancy, we examined the associations of FA supplementation, dietary folate intake, and RBC folate levels with fecundability. Several folate-rich foods were positively associated with RBC folate concentrations. However, in separate models, only seafood intake (1–3 times per week) was significantly associated with higher fecundability, while FA supplementation and RBC folate status were not. In joint models, positive associations were observed between FA supplementation and fecundability. Overall, these findings suggest that the association between folate and fecundability may be complex and encourage adequate folate intake during the preconception period.

Previous prospective cohort studies have reported positive associations between FA supplementation and fecundability. A study conducted in Singapore has reported that women who used FA supplements have higher fecundability compared with non-users (FR: 1.26, 95% CI: 1.03–1.56) [21]. Similarly, a prospective cohort study of 3895 Danish women planning pregnancy has shown that FA supplementation is associated with increased fecundability (FR:1.15, 95% CI: 1.06–1.25) [22]. In addition, higher folate intake, particularly from supplements, has been linked to improved reproductive outcomes, including higher ovarian reserve indicators and improved live birth rates following assisted reproductive technology treatment [28,29,30]. Several biological mechanisms may explain how folate could influence fertility. From a biological perspective, folate plays a central role in one-carbon metabolism, which is critical for DNA synthesis, repair, and methylation processes essential for oocyte quality, embryo development, and chromosomal stability [31]. Folate is also involved in homocysteine metabolism, and elevated homocysteine has been associated with oxidative stress, endothelial dysfunction, and impaired early embryonic development [32,33,34].

Despite these plausible mechanisms, epidemiological evidence remains inconsistent. Some studies, including systematic reviews and population-based analyses, have reported no significant associations between folate status and reproductive outcomes such as pregnancy rates, miscarriage, or assisted reproductive success [35,36,37,38,39]. Consistent with these studies, we did not observe a significant association between RBC folate status and fecundability after adjustment for potential confounders. Null findings have also been observed in specific populations, such as women with previous pregnancy loss and in folate-replete groups [23,24]. Results from large prospective cohort studies remain heterogeneous, with some suggesting modest associations between low dietary folate intake and reduced fecundability, but with inconsistent findings across populations [40]. Overall, the association between folate and fecundability appears complex.

Several factors may help explain our findings. First, the study was conducted during the early stage of FA supplementation policy implementation in China. Although FA supplementation showed associations with higher fecundability, its use in this cohort was low, limiting statistical precision. In addition, participants were recruited from women in northern China between 2009 and 2012. Therefore, the findings may not be fully generalizable to contemporary populations or to populations with different sociodemographic characteristics and FA supplementation practices. Second, the positive association with seafood intake may reflect overall dietary quality or socioeconomic factors, especially in Shanxi, where seafood consumption is generally low. Third, RBC folate concentrations were not associated with fecundability, which may be partly explained by relatively low and limited variability in folate levels in this population. Folate concentrations in this cohort were lower than those reported in populations with FA fortification and somewhat lower than levels observed in other regions of China [41,42], which may have reduced variability and the ability to detect associations. In addition, genetic variation, such as methylenetetrahydrofolate reductase (MTHFR) C677T polymorphism affecting folate metabolism, may modify the association between folate and fertility [43]. Furthermore, fecundability is influenced by multiple factors including age, ovarian reserve, BMI, male fertility, and environmental exposures, which may have stronger effects on TTP than folate status alone.

From a public health perspective, this study provides insight into folate-related exposures among women planning their first pregnancy during the early stage of FA promotion in China. The use of FA supplementation was low in this cohort, indicating that preconception supplementation was not yet widely adopted during the study period. Although our findings do not provide strong evidence for an independent association between FA supplementation, dietary folate intake, RBC folate status, and fecundability, they support further continued attention to adequate folate intake among women planning pregnancy because of its established benefits for maternal and fetal health. In particular, premarital health examinations may provide an important opportunity to deliver folate-related health education and encourage the initiation of supplementation among women preparing for pregnancy. Future studies incorporating repeated biomarker measurements, more detailed assessment of supplement use, and larger numbers of supplement users are needed to further clarify the role of folate in fecundability.

This study has several strengths. First, the prospective cohort design ensured that folate-related exposures were assessed before conception, thereby reducing recall bias and minimizing the possibility of reverse causation, whereby women experiencing prolonged difficulty conceiving might subsequently modify their diet or initiate nutritional supplementation. In addition, participants were recruited according to predefined eligibility criteria, and major demographic and lifestyle factors were adjusted for in multivariable analyses to reduce potential selection bias and confounding. Second, multiple dimensions of folate exposure, including supplementation, dietary intake, and biomarker-based folate status, were evaluated simultaneously, allowing a more comprehensive assessment. Third, the study focused on women without prior pregnancy, which minimized potential confounding from reproductive history and improved the homogeneity of the study population.

Several limitations should be acknowledged. First, folate-related exposures were assessed only at baseline, and no information was available on changes in diet or FA supplementation during follow-up. Detailed information on FA dosage and food portion sizes was not collected, preventing quantitative assessment of total folate intake. In addition, FA supplementation and dietary intake were assessed using questionnaires specifically developed for the original cohort study. These instruments have been widely used within this cohort, and the observed associations between dietary intake measures and RBC folate concentrations provide indirect support for their ability to capture relevant folate-related exposures. Nevertheless, some degree of measurement error and recall bias cannot be excluded. Second, although analyses were adjusted for major demographic and lifestyle factors, fecundability is influenced by multiple determinants. Some potentially relevant factors, including detailed male fertility characteristics, reproductive health conditions, environmental exposures, and active smoking, were not systematically available. Finally, participants were recruited from two counties in Shanxi Province during 2009–2012, when FA supplementation during the preconception period was relatively uncommon. Therefore, the findings may not be fully generalizable to contemporary populations or to settings with different sociodemographic characteristics, dietary patterns, and folate supplementation practices.

## 5. Conclusions

In this prospective cohort of reproductive age women, a comprehensive assessment of folate-related exposures showed limited evidence of an association between folate and fecundability. Positive associations for FA supplementation were observed considering dietary intake and folate status. These findings are broadly consistent with current recommendations promoting adequate folate intake during the preconception period. Overall, this study provides foundational evidence on folate-related exposures and fecundability in a preconception cohort from northern China. Further studies with repeated exposure assessment and larger numbers of supplement users are needed to clarify the role of folate in fecundability.

## Figures and Tables

**Table 1:** Baseline characteristics of the study population (*N* = 1475).

Variable	Frequency (%)
Age	
<25	1020 (69.2)
25–30	386 (26.2)
30–35	58 (3.9)
≥35	11 (0.7)
BMI (kg/m^2^)	
<18.5	147 (10)
18.5–24	961 (65.2)
≥24	356 (24.1)
Missing	11 (0.7)
Education	
Junior high school and below	868 (58.8)
High school and above	607 (41.2)
Occupation	
Farmer	726 (49.2)
Worker	117 (7.9)
Other *	632 (42.8)
Passive smoking	
No	906 (61.4)
Yes	569 (38.6)
Active smoking	
No	1465 (99.3)
Yes	10 (0.7)
Residence	
Rural	1022 (69.3)
Urban	452 (30.6)
Missing	1 (0.1)
Folic acid supplementation	
No	1432 (97.1)
Yes	24 (1.6)
Missing	19 (1.3)

* Other occupations included commercial workers, service personnel, technical staff, managerial personnel, and other occupations not classified elsewhere.

**Table 2:** Pregnancy rates within 1 and 2 years according to red blood cell folate status.

Red Blood Cell Folate Status	1-Year Pregnancy Rate				2-Year Pregnancy Rate			
	Pregnant	Total	Pregnancy Rate (%)	*p*	Pregnant	Total	Pregnancy Rate (%)	*p*
Deficiency (<340 nmol/L)	100	115	87.0	0.045	102	115	88.7	0.006
Non-deficiency (≥340 nmol/L)	919	995	92.4		941	991	95.0	

**Table 3:** Associations of individual folate-rich food intake frequencies with red blood cell folate concentrations: results from separate multivariable models.

Food	Frequency (/Week)	Estimate Effect	*p* for FDR Correction
Corn	<1 time	1	
	1–3 times	6.22 (1.16, 11.53)	0.077
	4–6 times	2.38 (−8.15, 14.13)	0.804
	≥7 times	11.40 (−3.22, 28.22)	0.367
Leafy vegetables	<1 time	1	
	1–3 times	−0.73 (−7.94, 7.04)	0.849
	4–6 times	3.86 (−3.81, 12.14)	0.513
	≥7 times	2.94 (−4.63, 11.12)	0.653
Solanaceous vegetables	<1 time	1	
	1–3 times	1.46 (−3.27, 6.41)	0.696
	4–6 times	3.04 (−2.09, 8.45)	0.501
	≥7 times	0.83 (−4.52, 6.48)	0.821
Root vegetables	<1 time	1	
	1–3 times	3.34 (−2.95, 10.04)	0.513
	4–6 times	8.65 (1.89, 15.86)	0.069
	≥7 times	9.42 (2.91, 16.35)	0.031
Fresh legumes	<1 time	1	
	1–3 times	2.11 (−1.21, 5.54)	0.462
	4–6 times	2.48 (−2.31, 7.50)	0.513
	≥7 times	4.58 (−2.03, 11.64)	0.447
Soy products	<1 time	1	
	1–3 times	−1.03 (−4.41, 2.46)	0.696
	4–6 times	3.32 (−1.78, 8.68)	0.462
	≥7 times	0.84 (−6.26, 8.47)	0.849
Seafood	<1 time	1	
	1–3 times	4.61 (0.20, 9.21)	0.152
	4–6 times	5.09 (−5.14, 16.43)	0.513
	≥7 times	2.06 (−10.14, 15.93)	0.821
Eggs products	<1 time	1	
	1–3 times	−1.62 (−6.44, 3.45)	0.696
	4–6 times	4.03 (−1.22, 9.55)	0.367
	≥7 times	0.96 (−4.40, 6.63)	0.821
Fruits	<1 time	1	
	1–3 times	4.92 (0.42, 9.63)	0.137
	4–6 times	12.77 (7.03, 18.83)	0.000
	≥7 times	8.01 (3.38, 12.85)	0.009
Nuts and seeds	<1 time	1	
	1–3 times	6.73 (2.30, 11.35)	0.026
	4–6 times	8.32 (−0.17, 17.52)	0.183
	≥7 times	3.56 (−3.65, 11.31)	0.513

Note: Each food was analyzed in a separate linear regression model. All models were adjusted for age group, BMI group, residence, education, occupation, passive smoking exposure, and folic acid supplementation. RBC folate levels were natural log-transformed. Effect size represents percent change in RBC folate calculated as (exp(β) − 1) × 100%. FDR: false discovery rate.

**Table 4:** Associations of folate-related exposures with time to pregnancy: results from separate multivariable discrete-time models.

Exposure Variables	n	FRs (95% CI)			
		≤6 Months	≤12 Months	≤24 Months	Overall
Folic acid supplementation					
No	1421	1	1	1	1
Yes	23	1.56 (0.91, 2.48)	1.28 (0.77, 1.99)	1.21 (0.73, 1.87)	1.27 (0.79, 1.93)
Dietary intake					
Corn					
<1 time/week	1240	1	1	1	1
1–3 times/week	175	0.88 (0.71, 1.09)	0.91 (0.75, 1.08)	0.96 (0.80, 1.14)	0.97 (0.81, 1.15)
4–6 times/week	25	1.10 (0.63, 1.76)	1.10 (0.68, 1.68)	1.12 (0.70, 1.68)	1.10 (0.69, 1.66)
≥7 times/week	16	1.14 (0.57, 2.01)	0.90 (0.48, 1.52)	0.93 (0.51, 1.54)	0.98 (0.55, 1.60)
Root vegetables					
<1 time/week	104	1	1	1	1
1–3 times/week	387	1.03 (0.78, 1.38)	0.97 (0.77, 1.23)	0.96 (0.76, 1.22)	0.97 (0.78, 1.24)
4–6 times/week	319	1.06 (0.80, 1.43)	0.96 (0.76, 1.23)	0.95 (0.75, 1.21)	0.96 (0.76, 1.23)
≥7 times/week	644	1.07 (0.82, 1.42)	0.95 (0.76, 1.20)	0.95 (0.76, 1.19)	0.96 (0.77, 1.21)
Seafoods					
<1 time/week	1202	1	1	1	1
1–3 times/week	201	1.29 (1.07, 1.55)	1.24 (1.05, 1.46)	1.23 (1.05, 1.44)	1.23 (1.04, 1.44)
4–6 times/week	32	1.19 (0.74, 1.79)	1.26 (0.85, 1.79)	1.28 (0.87, 1.81)	1.27 (0.86, 1.79)
≥7 times/week	21	0.85 (0.44, 1.47)	0.73 (0.42, 1.18)	0.85 (0.51, 1.31)	0.83 (0.50, 1.28)
Fruits					
<1 time/week	235	1	1	1	1
1–3 times/week	401	0.90 (0.73, 1.12)	0.98 (0.81, 1.17)	1.00 (0.83, 1.20)	0.99 (0.83, 1.19)
4–6 times/week	229	1.02 (0.80, 1.29)	1.06 (0.86, 1.31)	1.10 (0.90, 1.35)	1.08 (0.88, 1.33)
≥7 times/week	590	1.01 (0.83, 1.25)	1.08 (0.91, 1.29)	1.10 (0.92, 1.31)	1.08 (0.91, 1.29)
Nuts and seeds					
<1 time/week	1121	1	1	1	1
1–3 times/week	213	1.10 (0.90, 1.32)	1.11 (0.94, 1.30)	1.11 (0.95, 1.30)	1.10 (0.94, 1.29)
4–6 times/week	52	1.32 (0.92, 1.82)	1.11 (0.80, 1.50)	1.22 (0.90, 1.62)	1.21 (0.89, 1.61)
≥7 times/week	67	0.87 (0.61, 1.21)	0.95 (0.71, 1.24)	0.97 (0.73, 1.25)	0.96 (0.73, 1.24)
Dietary folate frequency score	1446	1.01 (0.98, 1.04)	1.01 (0.98, 1.04)	1.01 (0.99, 1.04)	1.01 (0.99, 1.04)
Red blood cell folate					
Deficiency (<340 nmol/L)	124	1	1	1	1
Non-deficiency (≥340 nmol/L)	1063	1.09 (0.85, 1.40)	1.13 (0.92, 1.40)	1.14 (0.93, 1.41)	1.14 (0.93, 1.41)

Note: Each exposure was analyzed in a separate multivariable discrete-time hazard model. Numbers do not sum to the total sample size because participants with missing values were excluded from each individual analysis. All models were adjusted for age group, BMI group, residence, education, occupation, and passive smoking exposure. FR: fecundability ratio; CI: confidence interval.

**Table 5:** Joint associations of folic acid supplementation, dietary folate frequency score, and red blood cell folate with time to pregnancy: results from multivariable discrete-time models.

Model	Exposure Variables	FRs (95% CI)			
		≤6 Months	≤12 Months	≤24 Months	Overall
Model 1	Folic acid supplementation				
	No	1	1	1	1
	Yes	1.73 (1.00, 2.76)	1.47 (0.88, 2.28)	1.39 (0.84, 2.16)	1.49 (0.92, 2.27)
	Dietary folate frequency score	1.01 (0.98, 1.05)	1.01 (0.98, 1.04)	1.01 (0.99, 1.04)	1.01 (0.99, 1.04)
Model 2	Folic acid supplementation				
	No	1	1	1	1
	Yes	2.07 (1.01, 3.77)	1.91 (1.00, 3.30)	1.91 (1.00, 3.30)	1.89 (0.99, 3.27)
	Dietary folate frequency score	1.01 (0.98, 1.04)	1.01 (0.98, 1.04)	1.01 (0.98, 1.04)	1.01 (0.98, 1.04)
	Red blood cell folate				
	Deficiency (<340 nmol/L)	1	1	1	1
	Non-deficiency (≥340 nmol/L)	1.09 (0.85, 1.41)	1.13 (0.92, 1.41)	1.13 (0.92, 1.41)	1.13 (0.92, 1.41)

Note: Model 1 includes folic acid supplementation and dietary folate score. Model 2 additionally includes red blood cell folate levels. All models were adjusted for age group, BMI group, residence, education, occupation, and passive smoking exposure. FR: fecundability ratio; CI: confidence interval.

## Data Availability

The data presented in this study are available on request from the corresponding authors due to participant privacy and ethical review board requirements.

## Abbreviations

The following abbreviations are used in this manuscript:

TTP	Time to pregnancy
FA	Folic acid
FR	Fecundability ratio
BMI	Body mass index
RBC	Red blood cell
Cls	Confidence intervals
